# Nicotinamide riboside intervention alleviates hematopoietic system injury of ionizing radiation‐induced premature aging mice

**DOI:** 10.1111/acel.13976

**Published:** 2023-08-31

**Authors:** Wenxuan Li, Xinyue Wang, Yinping Dong, Qidong Huo, Tongpeng Yue, Xin Wu, Lu Lu, Junling Zhang, Yu Zhao, Hui Dong, Deguan Li

**Affiliations:** ^1^ Tianjin Key Laboratory of Radiation Medicine and Molecular Nuclear Medicine, Institute of Radiation Medicine Chinese Academy of Medical Science & Peking Union Medical College Tianjin China

**Keywords:** cellular senescence, ionizing radiation, nicotinamide riboside, radiotherapy

## Abstract

Radiotherapy destroys cancer cells and inevitably harms normal human tissues, causing delayed effects of acute radiation exposure (DEARE) and accelerating the aging process in most survivors. However, effective methods for preventing premature aging induced by ionizing radiation are lacking. In this study, the premature aging mice of DEARE model was established after 6 Gy total body irradiation (TBI). Then the therapeutic effects and mechanism of nicotinamide riboside on the premature aging mice were evaluated. The results showed that 6 Gy TBI induced premature aging of the hematopoietic system in mice. Nicotinamide riboside treatment reversed aging spleen phenotypes by inhibiting cellular senescence and ameliorated serum metabolism profiles. Further results demonstrated that nicotinamide riboside supplementation alleviated the myeloid bias of hematopoietic stem cells and temporarily restored the regenerative capacity of hematopoietic stem cells probably by mitigating the reactive oxygen species activated GCN2/eIF2α/ATF4 signaling pathway. The results of this study firstly indicate that nicotinamide riboside shows potential as a DEARE therapeutic agent for radiation‐exposed populations and patients who received radiotherapy.

## Graphical abstract

The premature aging mice of delayed effects of acute radiation exposure (DEARE) model is established after 6 Gy total body irradiation. Nicotinamide riboside assists senescent spleen recovery in IR‐induced premature aging mice and ameliorated serum metabolism profiles. Nicotinamide riboside alleviates LSKs senescence probably by mitigating the ROS‐activated GCN2/eIF2α/ATF4 pathway in mice with IR‐induced premature aging.

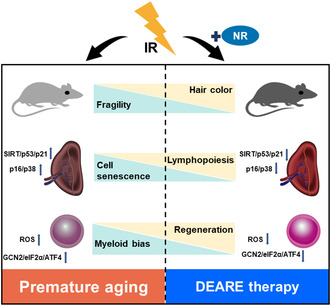

**Abbreviations**
ATF4: activating transcription factor 4
BM: bone marrow
BMNCs: bone marrow nucleated cells
CAT: catalase
CRA: competitive repopulation assay
DEARE: delayed effects of acute radiation exposure
eIF2α: eukaryotic translation initiation factor 2
GCN2: general control non‐derepressible 2
HSCs: hematopoietic stem cells
IR: ionizing radiation
LC‐MS: liquid chromatography‐mass spectrometry
MDA: malondialdehyde
NAD: nicotinamide adenine dinucleotide
NR: nicotinamide riboside
ROS: reactive oxygen species
TBI: total body irradiation

## 1 INTRODUCTION

Radiotherapy is one of the most common cancer treatment methods; however, it may cause morbidity and delayed effects of acute radiation exposure (DEARE) in a variety of organ systems, including the cardiovascular, gastrointestinal, pulmonary, neurological, and hematopoietic systems, which collectively contributes to accelerated aging (Cupit‐Link et al., 2017; Tung et al., 2021). Clinical studies have shown that an increasing number of cancer survivors with DEARE, including childhood and adult patients, become frail after diagnosis and treatment, thus leading to the development of chronic diseases and geriatric syndromes prematurely (Miller et al., 2022; Pranikoff et al., 2022). These changes are referred to as accelerated or premature aging in long‐term survivors of cancer. For example, the incidence of chronic health conditions among childhood cancer survivors is 73.4% (Oeffinger et al., 2006), and survivors estimated life expectancy is 30% lower than that of the general population (Shafqat et al., 2022). However, there are still no approved countermeasures against premature aging and DEARE induced by ionizing radiation (IR).

The hematopoietic system is extremely sensitive to IR. Moderate or high doses of IR induce bone marrow (BM) suppression, mainly due to the senescence of hematopoietic stem cells (HSCs) (Shao et al., 2013). Compared to young HSCs, senescent HSCs undergo phenotypic and functional changes, including reduced self‐renewal ability, impaired homing, and myeloid‐biased differentiation, which ultimately cause exhaustion of the regeneration function (Li et al., 2020). Oxidative stress induced by reactive oxygen species (ROS) is considered to be a prominent factor that triggers HSC senescence (Shao, Luo et al., 2014), and our previous studies have shown that IR induces persistent ROS elevation in HSCs (Chai et al., 2015; Lu et al., 2016).

General control non‐derepressible 2 (GCN2), a protein kinase sensor, is crucial for maintaining normal cellular growth and metabolism. When amino acids are lacking, GCN2 is activated by uncharged tRNA, which phosphorylates the α subunit of eukaryotic translation initiation factor 2 (eIF2α) (Hao et al., 2005; Ravindran et al., 2016). The translation of most mRNAs is reduced as a result of this phosphorylation, but some of them, including activating transcription factor 4 (ATF4), show an increase in translation (Carraro et al., 2010). GCN2/eIF2α/ATF4 plays crucial roles in apoptosis, proliferation, and differentiation, making it a major prospective therapeutic target for the treatment of many diseases (Chalvon‐Demersay et al., 2023; Croucher et al., 2021; Lee et al., 2023). Multiple studies have found that the GCN2‐eIF2α‐ATF4 axis is activated in various hematopoietic diseases, including acute lymphoblastic leukemia, acute myelocytic leukemia, and multiple myeloma (Nwosu et al., 2022). According to recent research, the GCN2‐eIF2α axis is an important component for HSCs that controls protein translation and energy consumption, and ATF4 deletion causes the expansion of phenotypical HSCs with functional attrition (Li et al., 2022; Sun, Lin, et al., 2021). However, its role in the IR‐induced premature aging of the hematopoietic system remains unclear.

Nicotinamide riboside (NR) is a pyridine‐nucleoside form of vitamin B3 that acts as a substrate for nicotinamide adenine dinucleotide (NAD^+^) and can effectively increase intracellular NAD^+^ levels. NAD^+^ is a critical coenzyme in energy metabolism and redox reactions that regulates the activity of sirtuins, poly ADP‐ribose polymerases (PARPs), and NADases (CD38, CD157, and SARM1) (Covarrubias et al., 2021). NR administration effectively restores NAD^+^ levels and ameliorates aging‐related stem cell dysfunction in the skeletal muscle and gut (Igarashi et al., 2019; Zhang et al., 2016). NR also exerts positive effects on HSCs, restoring their metabolic capacity by modifying mitochondrial function (Sun, Cao, et al., 2021; Vannini et al., 2019). However, the impact of NR treatment on IR‐induced premature aging in mice and whether it can rejuvenate HSC function requires further investigation.

In the present study, we investigated the DEARE therapeutic effects of NR using a mouse model of IR‐induced premature aging. We found that the administration of NR ameliorated the aging phenotypes of the spleen in IR‐induced prematurely aging mice by inhibiting SIRT1/p53/p21 and p38/p16. Moreover, our results indicated that NR treatment temporarily alleviated the myeloid bias of HSCs and restored the HSC regenerative capacity probably by mitigating the ROS‐activated GCN2/eIF2α/ATF4 pathway. NR first showed potential to be a DEARE therapeutic agent for patients receiving radiotherapy and exposed populations.

## 2 MATERIALS AND METHODS

### 2.1 Animals

Male C57BL/6 (CD45.2) mice were purchased from Beijing HFK Bioscience Co, Ltd. Male C57BL/6 (CD45.1) mice were purchased from Department of Laboratory Animal Science, Peking University Health Science Center (PKUHSC). The mice were used at the age of approximately 8–10 weeks. All animal experiments in our study were approved by the Animal Care and Ethics Committee at IRM‐CAMS (2022030).

### 2.2 Antibodies

Antibodies used in this paper was listed in Table S1.

### 2.3 Irradiation and treatment

The mice were divided into three groups: control, IR, and IR + NR. The mice in the IR and IR + NR groups were exposed to 6 Gy γ‐ray at a dose rate of 0.883 Gy/min (Gammacell‐40 ^137^Cs irradiator, Atomic Energy of Canada Ltd). Eight weeks after IR, the mice in the IR + NR group were administered NR via gavage at a dose of 400 mg/kg/d for 21 days. As a control, mice in the control and IR group received the same volume of solvent control for the same duration and frequency as those in the IR + NR group.

### 2.4 Running test

Mice were adapted to the running machine for 3 days prior to endurance testing by running at 10–15 m/min for 20 min. In endurance testing, the speed started at 5 m/min for 10 min at 5° inclination, and then the speed was increased by 1 m every minute until 16 m/min. Mice were considered exhausted when they rested on the plate for more than 10 s without attempting to continue running, and the time was recorded.

### 2.5 Quantification of NAD ^+^ levels

Quantification of relative NAD levels in serum was performed by the CoenzymeINAD(H) Content Assay Kit (BC5190) by Solarbio according to the manufacturer's instructions.

### 2.6 Quantification of malondialdehyde (MDA) levels

Quantification of relative MDA levels was performed on serum using the Malondialdehyde (MDA) assay kit (A003‐1‐2) by Nanjing Jiancheng Bioengineering Institute according to the manufacturer's instructions.

### 2.7 Analysis of enzymatic activity of catalase (CAT)

Enzymatic activity of CAT was performed on serum using the Catalase (CAT) assay kit (A007‐1‐1) by Nanjing Jiancheng Bioengineering Institute according to the manufacturer's instructions.

### 2.8 UPLC–MS analysis

The serum metabolites were separated and quantified by Ultra performance liquid chromatography‐Mass spectrum (UPLC–MS) as described previously (Yin et al., 2022).

### 2.9 Spleen index analysis

The spleen index was calculated as a percentage of spleen weight (g)/mice body weight (g).

### 2.10 SA‐β‐Gal staining

Spleen tissues were embedded into OCT (Sakura Finetek). The samples were sectioned into 6 μm sections using a cryostat (Leica Biosystems Nussloch GmbH). The senescence‐associated β‐galactosidase kit (Beyotime) was used to detect the senescence in spleen tissue and isolated LSKs following the manufacturer's protocols.

### 2.11 Western blotting

Spleen tissues were crushed and lysed first. After centrifugation at 12,000 rpm/min for 15 min, the concentration of total protein in the supernatant was detected using a BCA kit (Beyotime). Proteins were separated by SDS‐PAGE and transferred to PVDF membranes. After blocking with 5% milk, the membranes were incubated with primary antibodies at 4°C overnight, and then incubated with secondary antibodies at room temperature for 1 h. The protein signals were detected using an ECL reagent (Beyotime). Intensity of protein bands was quantitated using Image‐J software (National Institutes of Health).

### 2.12 RNA isolation and qPCR

Total RNA was extracted from spleen tissues using Trizol Reagent (Invitrogen) and reverse transcribed to cDNA using the 1st Strand cDNA Synthesis SuperMix (Yeasen Biotechnology). qPCR SYBR Green Master Mix (Yeasen Biotechnology) was applied to quantify the relative expression of mRNA. Primers used for quantitative PCR are listed in Table S2. The relative mRNA levels were analyzed according to the 2^−△△Ct^ method. Each experiment was performed in triplicate.

### 2.13 Peripheral blood cell count

Blood was obtained from the mice via the orbital sinus and was collected in a micro‐pipette coated with an anticoagulant and analyzed on a Celltac E hematology analyzer (Nihon kohden). The following measurements were recorded: white blood cell (WBC), red blood cell (RBC), platelet (PLT) counts, and the percentage of neutrophil (NE%), lymphocyte (LY%), monocyte (MO%), and eosinophil (EO%).

### 2.14 Flow cytometry analysis

To measure CD4^+^ T cells, CD8^+^ T cells, and B cells in spleen, 1 × 10^6^ spleen cells were stained with CD4, CD8, and B220 at room temperature (RT). To measure neutrophil, monocyte, and eosinophil in peripheral blood, peripheral blood cells were stained with CD45, CD170, CD115, CD172a, CD11b, Ly‐6G, and Ly‐6C after the red blood cells were removed with the 0.15 mol/L NH4Cl solution. BM cells were isolated by flushing both the tibias and femurs with sterile PBS, and the number of cells was counted by the hemocytometer. To measure B cells, T cells, and myeloid cells in BM, 1 × 10^6^ cells were stained with B220, CD3, CD11b, and Gr1 at RT. To analyze the frequencies of Lin^−^Scal^+^c‐Kit^+^ cells (LSKs), hematopoietic progenitor cells (HPCs), common lymphatic progenitor cells (CLPs), common myeloid progenitor cells (CMPs), granulocyte‐macrophage progenitor cells (GMPs), and megakaryocyte erythroid progenitor cells (MEPs) in bone marrow, 5 × 10^6^ BM cells were stained with biotin‐conjugated lineage antibodies for surface markers and then stained with streptavidin, c‐kit, Sca‐1, CD127, CD135, CD34, and CD16/32. To probe the ROS level, BM cells were stained for LSKs and HPCs and then incubated with 10 μM DCF‐DA for 20 min at 37°C. For the analysis of p‐GCN2, p‐eIF2α, and ATF4, BM cells were processed as described for the experiments with LSKs, fixed and permeabilized with eBioscience Foxp3/Transcription Factor Staining Buffer Set (Invitrogen) before being stained with anti‐p‐GCN2 (Thr898), anti‐p‐eIF2α (Ser51), or anti‐ATF4 antibody. The samples were detected by BD FACS Celesta (BD Bioscience) and analyzed by FlowJo software.

### 2.15 Isolation of LSKs and immunofluorescence analysis

BM cells were isolated as described above, and were incubated with biotin‐conjugated lineage antibodies for surface markers and then stained with streptavidin, c‐kit and Sca‐1. LSKs were sorted using a BD Aria FACS II cell sorter (BD Bioscience) and put on the slide, before fixed using 4% paraformaldehyde at 4°C. Then LSKs were washed with PBS and permeabilized using 1% Triton X‐100 for 15 min at RT. LSKs were then blocked with 5% goat serum at RT for 1 h and incubated with anti‐p‐GCN2 (Thr898) (1:100) or anti‐ATF4 (1:100) antibody at 4°C overnight, followed by alexa‐488 conjugated anti‐rabbit secondary antibody (1:200) or CoraLite Plus 488‐conjugated p‐eIF2α (Ser51) antibody (1:100) at 37°C for 1 h. Cells were again washed twice and stained with DAPI (10 μg/mL, Solarbio) and then observed by a confocal laser scanning microscope (CLSM) (Nikon, C2) using the 60× objective. The intracellular fluorescence signal intensity was analyzed with Image‐J software (National Institutes of Health).

### 2.16 Competitive repopulation assay (CRA)

In the present study, donor cells (2 × 10^6^ BM cells) were collected from C57BL/6 (CD45.2) mice. Then the donor cells were transplanted into the lethally irradiated (9.0 Gy) C57BL/6 (CD45.1) recipient mice through lateral canthus vein injection. For analysis of engraftment, the percentage of donor‐derived (CD45.2^+^) cells in the peripheral blood recipients was examined 1 and 2 months after transplantation. The bone marrow cells were examined 2 months after transplantation. The samples were stained with CD45.1, CD45.2, B220, CD3, CD11b, and Gr‐1. The cells were calculated and analyzed with BD FACS Celesta (BD Bioscience).

### 2.17 Statistical analysis

Statistical analysis was performed using GraphPad Prism 8 software. The data were expressed as means ± SD, and results with *p* < 0.05 were considered statistically significant. Error bars represent the standard error of the mean in all figures.

## 3 RESULTS

### 3.1 NR relieved IR‐induced premature aging in mice

**FIGURE 1:**
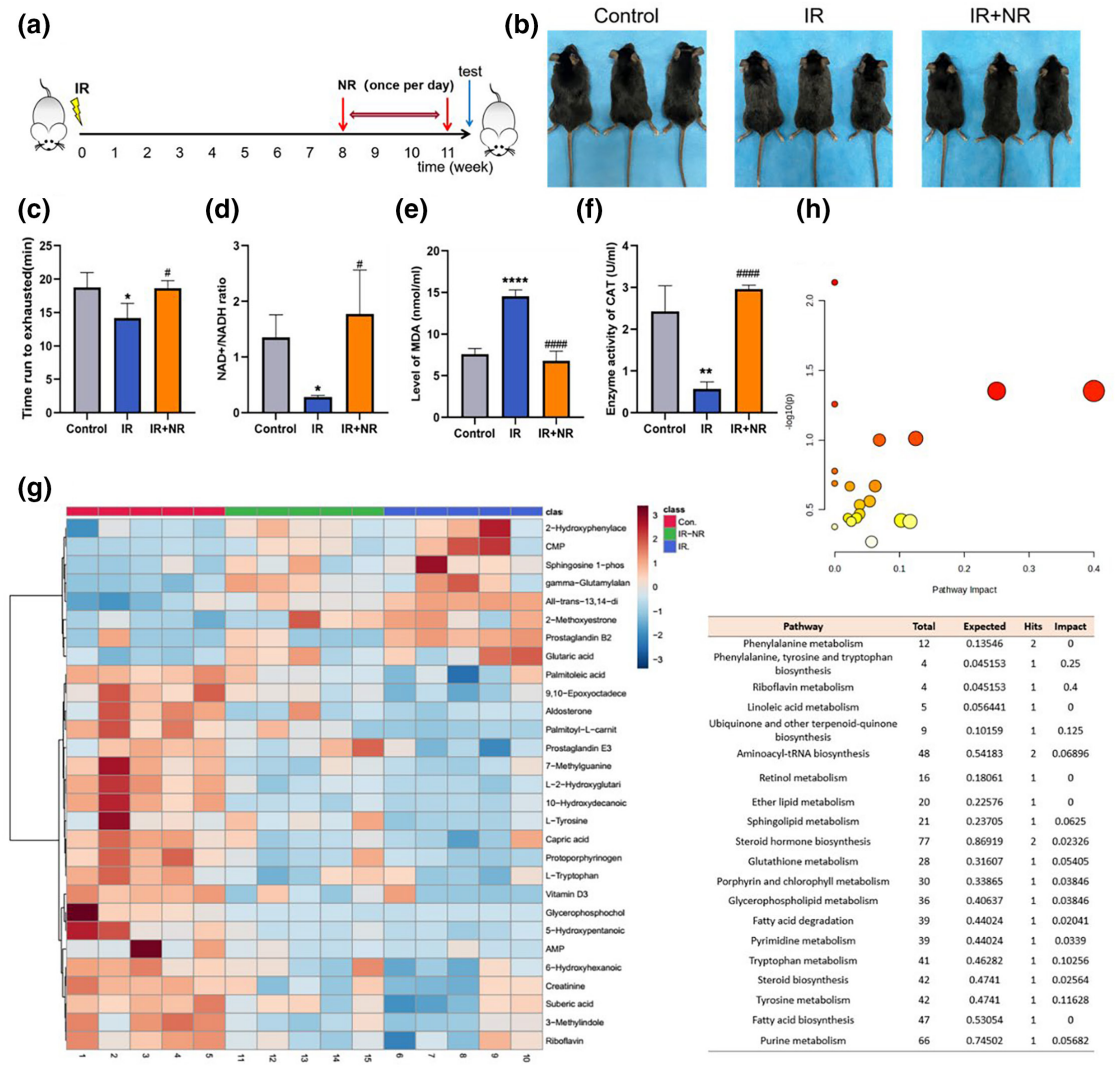
NR relieved IR‐induced premature aging in mice. (a) Schematic of the experimental procedure for mice experiencing IR and NR intervention; (b) Snapshot comparison of mice from control group, IR group, and IR + NR group, respectively (*n* = 3); (c) Measurement of running distance on the treadmill of experimental mice (*n* = 4); (d) Quantification of relative NAD^+^ levels in serum (*n* = 3); (e) Quantification of MDA levels in serum (*n* = 4); (f) Enzyme activity of CAT in serum (*n* = 4); (g) Clustering heat map analysis of differential metabolites identified among three groups (*n* = 5); (h) KEGG pathway analysis of differential metabolites among three groups. Data in bar graphs were shown as mean ± SD. Statistical analyses were performed using unpaired *t*‐test, *p < 0.05 versus Control, **p < 0.01 versus Control. ****p < 0.0001 versus Control; ^#^ *p* < 0.05 versus IR, ^####^ *p* < 0.0001 versus IR.

The irradiated mice experienced premature aging as documented by their premature frailty, decreased cognitive competence, and increased mortality (Fielder et al., 2019). In this study, male C57BL/6 mice were exposed to 6 Gy total body irradiation (TBI), followed by treatment with NR (400 mg/kg/day via oral gavage) or a vehicle 8 weeks after IR, according to the schedule (Figure 1a). Mice were observed and assessed 11 weeks after IR. We observed the hair of irradiated mice turned gray and lost its luster, which was largely reversed by NR administration (Figure 1b). Moreover, IR significantly compromised the exercise capacity of the mice, as measured by treadmill endurance testing. NR intervention provided substantial benefits for exercise capacity (Figure 1c). As reported previously, some tissues of aging mice show decreased levels of NAD^+^ (Camacho‐Pereira et al., 2016). Our results showed that IR markedly reduced the level of NAD^+^ in mouse serum (Figure 1d). Additionally, the increased levels of MDA and impaired antioxidant enzyme activity of CAT in the serum indicated that IR exacerbated oxidative stress in mice (Figure 1e,f). Oral administration of NR to irradiated mice for 3 weeks greatly boosted NAD^+^ levels, reduced the production of MDA, and improved the enzyme activity of CAT in the serum (Figure 1d–f).

Serum metabolite profiles were analyzed using liquid chromatography–mass spectrometry (LC–MS). The clustering heat map showed a total of 29 differential metabolites in the serum among the three groups, of which eight metabolites were upregulated and 21 metabolites were downregulated after IR, mainly amino acids, steroids, and organic acids (Figure 1g). The differential metabolism data of each group of samples were uploaded to the Kyoto Encyclopedia of Genes and Genomes (KEGG) database for pathway analysis, and the results showed that the differential metabolites were mainly enriched in the riboflavin metabolism, amino acid biosynthesis and metabolism, purine metabolism, and glutathione metabolism pathways (Figure 1h). Based on these results, we preliminarily concluded that 6 Gy TBI induced premature aging in mice and that the indicators of premature aging were relieved when mice were treated with NR 8 weeks after IR.

### 3.2 NR alleviated premature aging of the spleen

**FIGURE 2:**
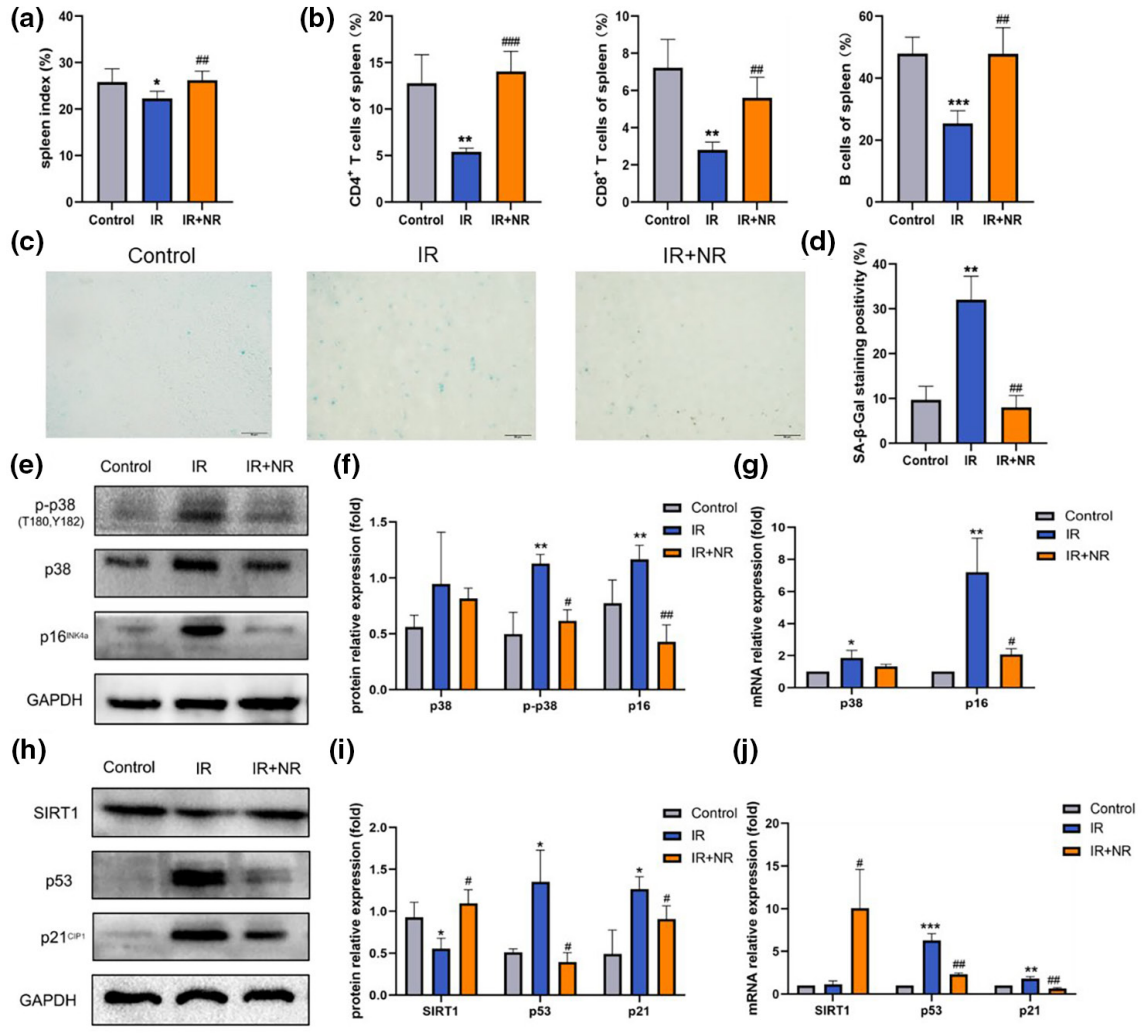
NR alleviated premature aging of the spleen. (a) The spleen index of mice (*n* = 5); (b) The percentages of CD4^+^ T cells, CD8^+^ T cells, and B cells in spleen (*n* = 4) (c) Representative images of SA‐β‐Gal staining of spleen of mice (scale bar: 50 μm); (d) Comparative statistics of SA‐β‐Gal staining of spleen (*n* = 3); (e, f) Protein expressions of phosphorylated p38, p38 and p16^INK4a^ in spleen which measured by western blotting and their quantification analysis; (g) Quantification analysis of mRNA expression of p38 and p16^INK4a^ (*n* = 3); (h, i) Protein expressions of SIRT1, p53 and p21 ^CIP1^ in spleen and their quantification analysis; (j) Quantification analysis of mRNA expression of SIRT1, p53 and p21 ^CIP1^ (*n* = 3). The results are expressed as the means ± SD. Statistical analyses were performed using unpaired *t*‐test, *p < 0.05 versus Control, **p < 0.01 versus Control, ***p < 0.001 versus Control; ^#^ *p* < 0.05 versus IR, ^##^ *p* < 0.01 versus IR.

The spleen is involved in immunization and stress hematopoiesis, in concert with the BM (Zhang et al., 2022). Because of its leukocyte activity and blood storage/filtration, the spleen is highly sensitive to IR. In the present study, we found that the spleen index of mice in the IR group decreased noticeably and that NR treatment restored the spleen index to normal levels (Figure 2a). Then, we analyzed lymphoid components in spleen and found that NR significantly elevated the level of T cells (both CD4^+^ T cells and CD8^+^ T cells) and B cells in spleen of irradiated mice during aging (Figure 2b). In addition, the spleen showed senescence with an increased SA‐β‐Gal staining percentage in the IR group. With NR treatment, the percentage of SA‐β‐Gal positive cells was significantly reduced (Figure 2c,d). These data suggest that NR ameliorated IR‐induced spleen senescence in mice.

Cellular senescence is caused by replicative or stress‐related senescence, with the activation of p53 and p16^INK4a^, respectively, leading to the activation of p21^CIP1^ and cell cycle arrest. Elevated expression of phosphorylated p38, p38, and p16^INK4a^ proteins in the spleen was observed in mice of the IR group. NR markedly reversed the effects of IR by inhibiting phosphorylated p38, p38, and p16^INK4a^ expression (Figure 2e,f). The patterns of change in p38 and p16^INK4a^ mRNA expression were the same as those of their proteins (Figure 2g). SIRT1 is an NAD^+^‐dependent deacetylase that inhibits the transcriptional activity of p53 (Ong & Ramasamy, 2018). SIRT1 expression in the spleen was decreased by IR, with increased expression of p53 and p21^CIP1^. When treated with NR, the proteins showed an inverse expression level compared to that in the IR group (Figure 2h,i). In addition, NR administration inhibited the IR‐induced upregulation of p53 and p21^CIP1^. Further, although the mRNA expression of SIRT1 was not attenuated by IR, NR significantly increased SIRT1 mRNA expression (Figure 2j). All results revealed that the spleen exhibited aging phenotypes in IR‐induced premature aging mice, and NR alleviated cellular senescence induced by IR via the p38/p16 ^INK4a^ and SIRT1/p53/p21 ^CIP1^ pathways.

### 3.3 NR ameliorated the imbalance of myeloid‐lymphoid differentiation

**FIGURE 3:**
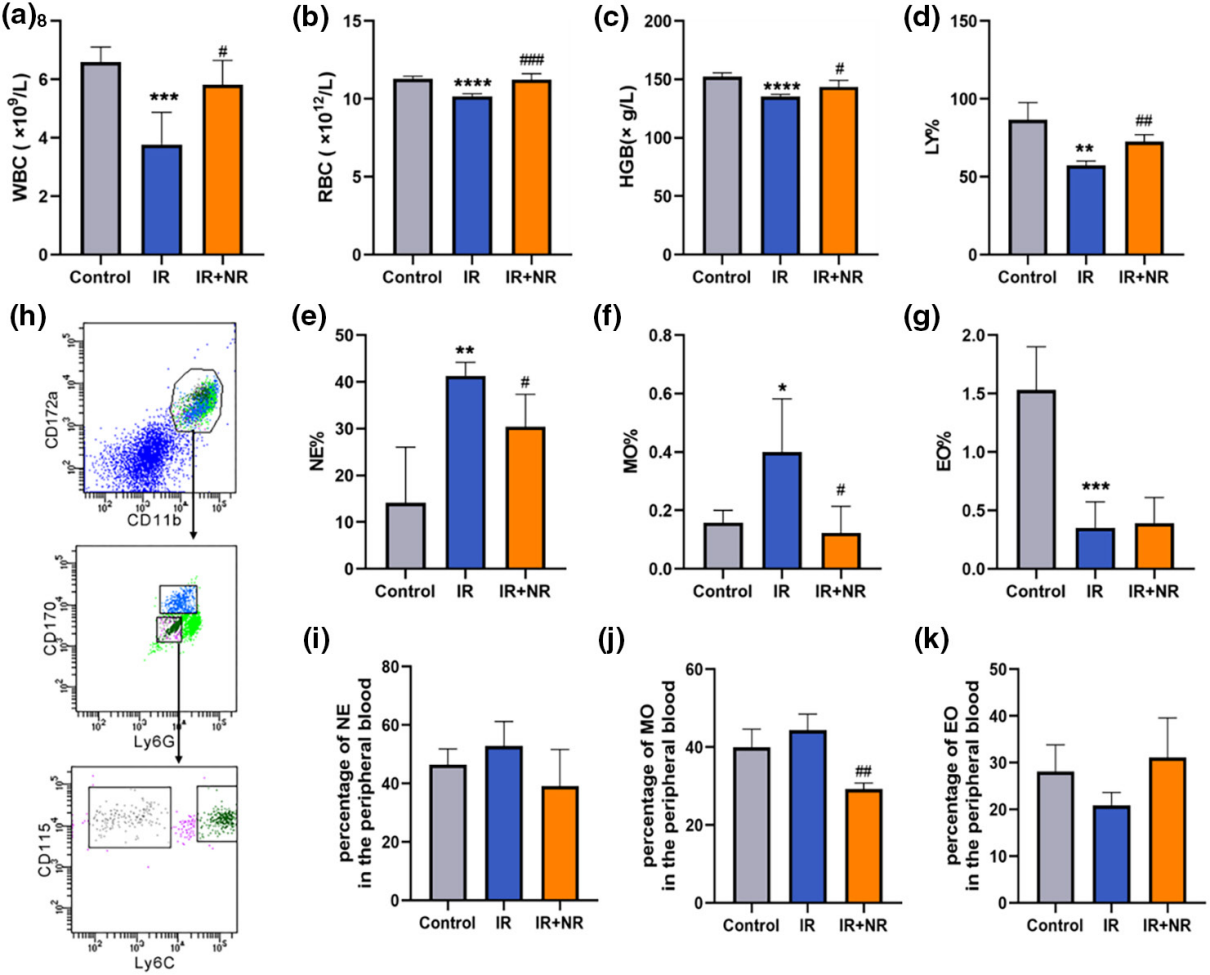
NR ameliorated the imbalance of myeloid‐lymphoid differentiation. (a–d) The numbers of (a) white blood cells (WBC) and (b) red blood cells (RBC), (c) the level of hemoglobin (HGB), and (d) the percentages of lymphocyte (LY) were analyzed in the peripheral blood (*n* = 5); (e–g) The percentage of (e) neutrophilic granulocytes (NE), (f) monocytes (MO) and (g) eosinophils (EO) were analyzed in the peripheral blood (*n* = 5); (h) A representative gating strategy of EO, MO and NE in the peripheral blood analyzed by flow cytometry; (i–k) The percentages of (i) NE, (j) MO and (k) EO in the peripheral blood were analyzed by flow cytometry (*n* = 4). Data in bar graphs were shown as mean ± SD. Statistical analyses were performed using unpaired *t*‐test, *p < 0.05 versus Control, **p < 0.01 versus Control, ***p < 0.001 versus Control, ****p < 0.0001 versus Control; ^#^ *p* < 0.05 versus IR, ^##^ *p* < 0.01 versus IR, ^###^ *p* < 0.001 versus IR.

To investigate how IR‐induced premature aging affects the hematopoietic system, the number of peripheral blood cells was analyzed 11 weeks after mice were exposed to 6 Gy TBI. Compared to control mice, mice receiving 6 Gy TBI showed decreased numbers of white blood cells (WBC), red blood cells (RBC), lymphocytes (LY), and hemoglobin (HGB). However, NR treatment reversed the decrease in WBC, RBC, HGB, and LY levels in the peripheral blood (Figure 3a–d). At the same time, significant increases were observed in the percentages of neutrophils (NE) and monocytes (MO), along with a decreased percentage of eosinophils (EO) in mice that received 6 Gy TBI (Figure 3e–g). The results of flow cytometry were similar (Figure 3h–k). NR administration alleviated these changes in the peripheral blood, especially by modifying the proportion of lymphocytes and myeloid‐derived cells, including NE and MO. These data suggest that IR‐induced premature aging could cause an imbalance in myeloid‐lymphoid differentiation in the peripheral blood, and treatment with NR effectively ameliorated the alterations in peripheral blood cells in prematurely aging mice.

### 3.4 NR affected bone marrow hematopoietic differentiation

**FIGURE 4:**
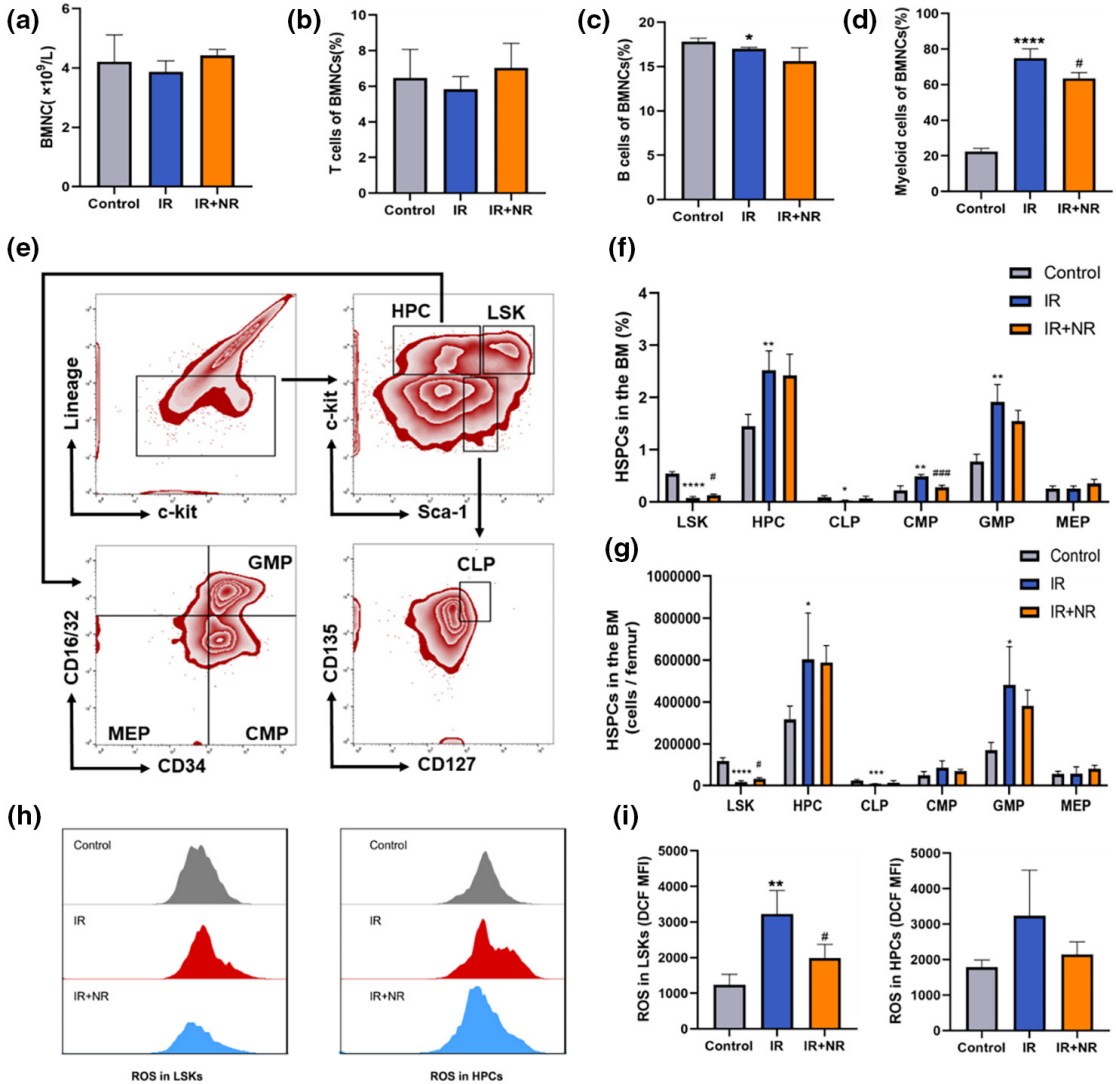
NR affected the bone marrow hematopoietic differentiation. (a) The number of BMNCs (*n* = 4); (b–d) The percentages of (b) T, (c) B, and (d) myeloid cells of BMNCs (*n* = 3); (e) Representative flow cytometric dot plots for LSK (Lin^−^Sca‐1^+^c‐Kit^+^), HPCs (Lin^−^Sca‐1^−^c‐Kit^+^), CLPs (Lin^−^Sca‐1^−^c‐Kit^−^CD127^+^ CD135^+^), CMPs (Lin^−^Sca‐1^−^c‐Kit^+^CD34^+^CD16/32^−^), GMPs (Lin^−^Sca‐1^−^c‐Kit^+^CD34^+^CD16/32^+^), and MEPs (Lin^−^Sca‐1^−^c‐Kit^+^CD34^−^CD16/32^−^) in the BM; (f) Frequencies of LSK, HPCs, CLPs, CMPs, GMPs, and MEPs which analyzed by flow cytometry (*n* = 4); (g) Numbers of LSK, HPCs, CLPs, CMPs, GMPs, and MEPs which analyzed by flow cytometry (*n* = 4); (h) Representative analysis of ROS level in LSKs and HPCs by flow cytometry; (i) The MFI of ROS level in LSKs and HPCs (*n* = 3). The results are expressed as the means ± SD. Statistical analyses were performed using unpaired *t*‐test, *p < 0.05 versus Control, ****p < 0.0001 versus Control; ^#^ *p* < 0.05 versus IR, ^##^ *p* < 0.01 versus IR.

Owing to their capacity for self‐renewal and differentiation, hematopoietic stem cells (HSCs) are crucial for maintaining homeostasis of the hematopoietic system; however, HSCs are also vulnerable to external stresses (Montazersaheb et al., 2022). In the present study, compared to control mice, mice that received 6 Gy TBI showed reduced numbers of bone marrow nucleated cells (BMNCs), T cells, and B cells, and an increased number of myeloid cells. After NR treatment, the number of myeloid cells significantly decreased, but the other cells showed little change (Figure 4a–d). To further examine the effect of IR on the hematopoietic system, LSKs and other immature hematopoietic cells in the BM were analyzed by flow cytometry, as shown in Figure 4e. The level of LSKs in mice exposed to 6 Gy TBI was markedly reduced, whereas NR treatment led to a significant increase (Figure 4f,g). In addition, the percentages of other progenitor populations, including HPCs, CLPs, CMPs, GMPs, and MEPs, all derived from HSCs, were altered by IR. A decrease in CLPs and an augmentation of GMPs, which indicated myeloid lineage bias, were observed in mice that received 6 Gy TBI (Figure 4f,g). Interestingly, NR partially decreased the skewing of myeloid and lymphoid lineage cells at the progenitor level with a considerable increase in the frequency of CLPs (Figure 4f,g) and a significant decrease in the frequency of GMPs (Figure 4j).

TBI can lead to long‐term BM suppression by specifically inducing high levels of intracellular ROS in LSKs. Therefore, we investigated whether NR reduces hematopoietic damage by decreasing ROS levels. ROS levels in the hematopoietic cells were measured using flow cytometry (Figure 4h). Compared with those in control mice, the ROS levels in the LSKs of irradiated mice were significantly elevated and were significantly decreased by NR treatment. However, the levels of ROS in the HPCs did not change significantly (Figure 4i). These data suggest that IR impairs the differentiation of HSCs and causes myeloid lineage bias. Oral administration of NR attenuated changes in BM hematopoietic cells, partially by relieving persistent oxidative stress in LSKs.

### 3.5 NR attenuated IR‐induced LSKs senescence

**FIGURE 5:**
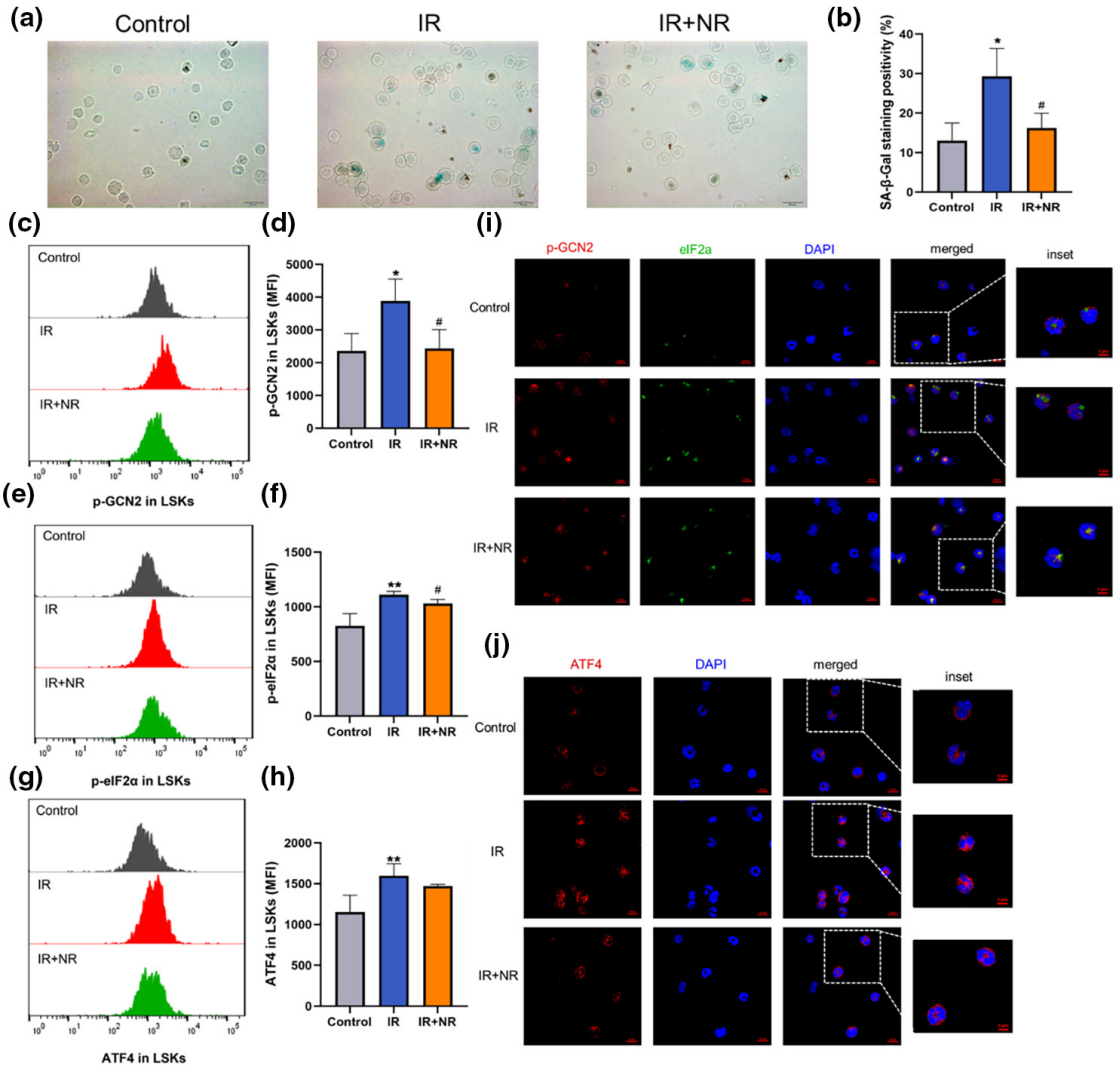
NR attenuated the LSKs senescence. (a) Representative images of SA‐β‐Gal staining of LSKs (scale bar: 20 μm); (d) Comparative statistics of SA‐β‐Gal staining of LSKs (*n* = 3); (c–h) Representative analysis of (c) p‐GCN2, (e) p‐eIF2α, and (g) ATF4 in LSKs, and the MFI of (d) p‐GCN2, (f) p‐eIF2α, and (h) ATF4 in LSKs detected by flow cytometry (*n* = 4); (i) Representative images of p‐GCN2 and p‐eIF2α in LSKs (red, p‐GCN2; green, p‐eIF2α; blue, DAPI); (j) Representative images of ATF4 in LSKs (red, ATF4; blue, DAPI), Scale bars: 10 or 5 mm (inset). The results are expressed as the means ± SD. Statistical analyses were performed using unpaired *t*‐test, *p < 0.05 versus Control, **p < 0.01 versus Control; ^#^ *p* < 0.05 versus IR, ^##^ *p* < 0.01 versus IR.

Exposure to IR induces long‐term hematopoietic injury by selectively inducing stem cell senescence. According, we isolated LSKs and assessed the senescence level by SA‐β‐gal staining (Figure 5a,b). The results showed that the expression of β‐galactosidase was increased in the LSKs of irradiated mice. NR treatment reduced the expression of β‐galactosidase in LSKs, which indicated that LSKs senescence was inhibited by NR treatment. To determine the underlying mechanism, flow cytometry and immunofluorescence were used to detect the expression of the GCN2/eIF2α/ATF4 axis in LSKs. As shown in Figure 5c–f, activated GCN2 (p‐GCN2) and phosphorylated eIF2α (p‐eIF2α) were significantly upregulated in LSKs of the IR mice group, and NR treatment largely inhibited the activation of GCN2 and eIF2α. The expression of ATF4 was subsequently increased in the LSKs of irradiated mice and was similarly downregulated by NR administration (Figure 5g,h). Confocal images also showed that the GCN2/eIF2α/ATF4 axis was activated by 6 Gy TBI, and these effects were inhibited by NR treatment (Figure 5i,j). These results suggest that NR attenuates LSKs senescence triggered by IR, possibly through GCN2/eIF2α/ATF4 axis.

### 3.6 NR enhanced the multilineage engraftment and reconstitution potential of HSCs

**FIGURE 6:**
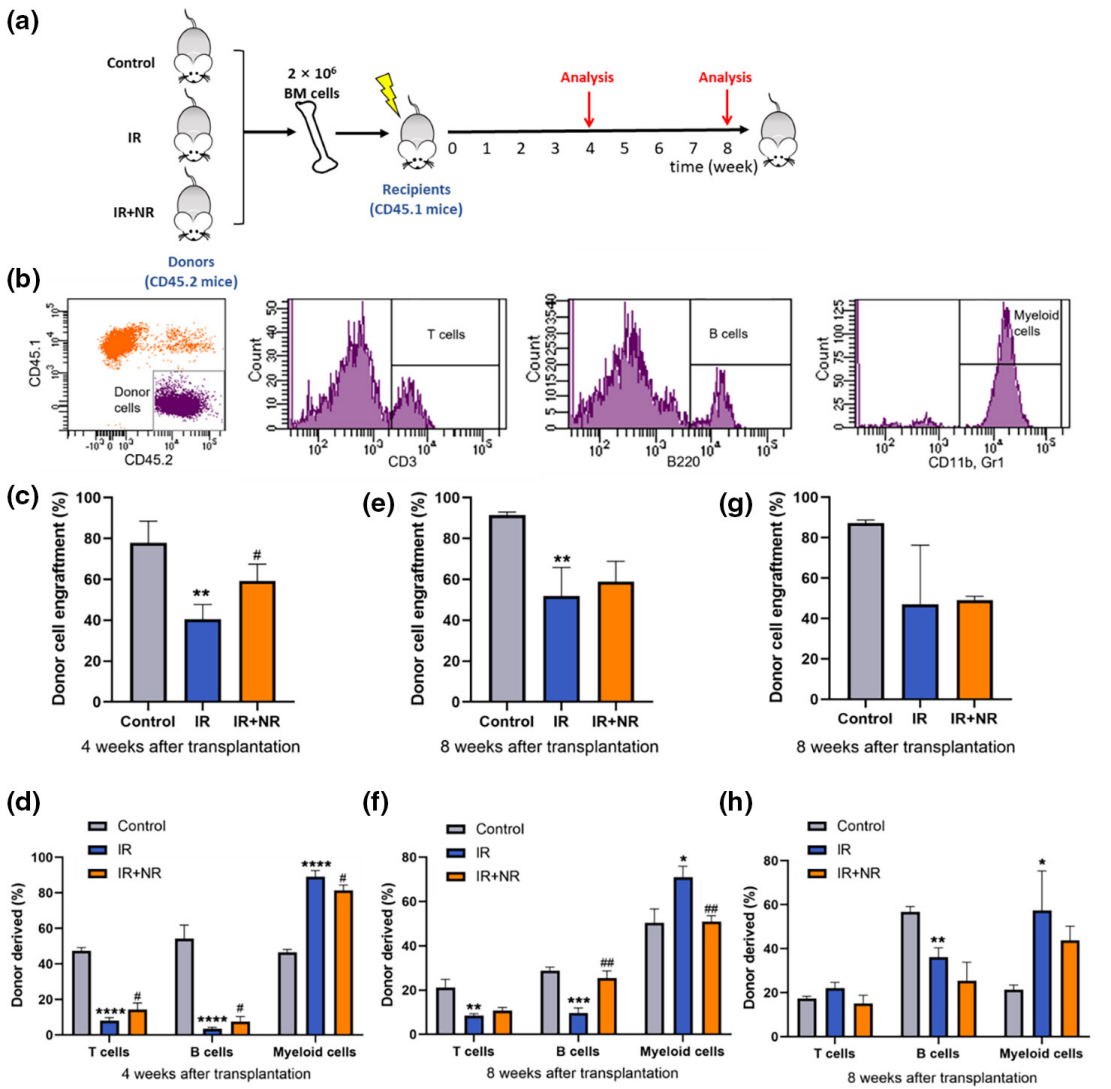
NR enhanced the multilineage engraftment and reconstitution potential of HSCs. (a) Scheme of the strategy for quantifying HSCs multilineage engraftment and reconstitution potential by competitive repopulation assay. (b) Representative flow cytometry plots showing percentage of donor (CD45.1^+^) cell engraftment, donor‐derived T cells (CD45.1^+^ CD3^+^), B cells (CD45.1^+^ B220^+^), and myeloid cells (CD45.1^+^ CD11^+^/Gr‐1^+^); (c) Donor‐derived cell multilineage engraftments in peripheral blood at 4 weeks after transplantation in lethally irradiated recipients; (d) The percentage of donor‐derived T, B, and myeloid cells in peripheral blood at 4 weeks after transplantation in lethally irradiated recipients; (e) Donor‐derived cell multilineage engraftments in peripheral blood at 8 weeks after transplantation in lethally irradiated recipients; (f) The percentage of donor‐derived T, B, and myeloid cells in peripheral blood at 8 weeks after transplantation in lethally irradiated recipients. (g) Donor‐derived cell multilineage engraftments in BM at 8 weeks after transplantation in lethally irradiated recipients; (h) The percentage of donor‐derived T, B, and myeloid cells in BM at 8 weeks after transplantation in lethally irradiated recipients. Results are presented as mean ± SD (*n* = 3). Statistical analyses were performed using unpaired *t*‐test, **p < 0.01 versus Control, ***p < 0.001 versus Control, ****p < 0.0001 versus Control; ^#^ *p* < 0.05 versus IR, ^##^ *p* < 0.01 versus IR.

Since our results suggest that IR affects the renewal and differentiation capacity of HSCs, it was essential to explore whether HSC function was compromised. Long‐term multilineage engraftment is the gold standard for measuring HSC function; thus, competitive repopulation assays (CRA) were performed (Figure 6a). Donor‐derived cell lineage engraftment in the peripheral blood of recipient mice, including T, B, and myeloid cells, was measured 4 or 8 weeks after transplantation (Figure 6b). As expected, the mice that received donor cells from control mice exhibited a high percentage of donor cell reconstitution, indicating successful engraftment. HSCs from irradiated mice exhibited significantly reduced reconstitution capacity, whereas NR treatment increased the reconstitution capacity (Figure 6c). Specifically, mice that received donor cells from irradiated mice showed a dramatic decrease in donor‐derived T and B cells, as well as an increased number of myeloid cells in the peripheral blood 4 weeks after transplantation, while NR treatment ameliorated these alterations (Figure 6d). The beneficial effects of NR were also observed in HSCs after 8 weeks of observations (Figure 6e). NR treatment preserved the multilineage engraftment of HSCs in irradiated mice, coupled with improved lymphocyte (T and B cells) and reduced myeloid cells (Figure 6f). We subsequently analyzed the changes in the BM, and the results showed that NR did not significantly improve the proportion of donor‐derived cells and multilineage engraftment of HSCs in bone marrow (Figure 6g,h). These data suggest that NR enhances the multilineage engraftment and reconstitution potential of HSCs in IR mice for a period of time.

## 4 DISCUSSION

Radiation therapy destroys cancer cells, but inevitably harms normal human tissues, causes DEARE, and accelerates the aging process for most survivors. However, there are relatively few studies on IR‐induced premature aging, and almost no corresponding measures to date (Kim et al., 2023). As a booster of NAD^+^, NR has been shown to reverse stem cell dysfunction in aged mice and plays a therapeutic role in a variety of aging‐related diseases, including Alzheimer's disease and diabetes (Igarashi et al., 2019; Lee & Yang, 2019; Xie et al., 2019). Moreover, NR exhibits antitumor effects, indicating that radiotherapy‐related damage can be safely treated (Hamity et al., 2020). In this study, we evaluated the therapeutic effects of NR on the hematopoietic system of prematurely aged mice and explored the underlying mechanisms. Our results demonstrated that NR ameliorated the aging phenotypes in our mouse model. Furthermore, NR treatment alleviated LSKs senescence and temporarily restored HSCs regenerative capacity probably by inhibiting the ROS‐activated GCN2/eIF2α/ATF4 axis.

First, the therapeutic effects of NR on IR‐induced aging phenotypes were observed. The results showed that abnormal body appearance, degenerated exercise capacity, and senescent spleen cells were attenuated by the administration of NR. Cellular senescence is mostly characterized by permanent cellular proliferation arrest and multiple phenotypic changes in which the sirtuin family of proteins plays key roles (Wiley & Campisi, 2021). SIRT1 is a NAD^+^‐dependent deacetylase that plays critical roles in a wide range of biological processes, including metabolism, energy balance, immune responses, and aging (Hall et al., 2013). A previous study showed that SIRT1 inhibits p53 transcription by deacetylating it at K382 in the C‐terminus and decreasing the expression of p21 in human endothelial progenitor cells (Lamichane et al., 2019). Our results showed that NR downregulated the expression of the p38/p16 ^INK4a^ and SIRT1/p53/p21 ^CIP1^ axis, thereby ameliorating cellular senescence in the spleen.

Hematopoiesis is a continuous process in which blood cells are produced, maintained at normal levels, and increase in response to demand (Li et al., 2020). TBI causes long‐term mouse BM injury via the induction of HSC senescence, which is mainly manifested as reduced self‐renewal activity, myeloid skewing, and impacted engraftment potential (Li et al., 2020; Shao, Feng, et al., 2014). In this study, WBC, RBC, neutrophils, lymphocytes, monocytes, and eosinophils underwent various changes after 6 Gy TBI. We subsequently detected hematopoietic cells in the BM and found an increased percentage of myeloid cells and a decreased percentage of lymphocytes. Their progenitor cells also showed the same changes, which indicated the myeloid lineage bias of HSCs in IR‐induced premature mice. NR showed a significant inhibitory effect on IR‐induced augmentation of myeloid cells, thereby improving myeloid skew in IR‐induced premature mice. The CRA results showed that the HSCs in irradiated mice exhibited significantly reduced reconstitution capacity. We expected NR to effectively reduce IR‐induced HSCs senescence and its physiological consequences, and our results confirmed this expectation at 4 weeks of transplantation. However, this effect did not extend to the eighth week, and the proportion of donor‐derived cells in BM was not elevated either. Therefore, we assume that the NR acts mainly in MPP, not HSCs. A recent review assesses the currently published research articles on human NR supplementation and claims that oral NR supplementation has displayed few clinically relevant effects (Damgaard & Treebak, 2023), which is consistent with our results. Specialized BM microenvironments, termed HSC niches, maintain HSC numbers and function through cellular interactions and secreted factors that can be damaged by IR (Morrison & Scadden, 2014). HSCs from NR‐treated, irradiated mice had limited ameliorative effects on cell engraftment, and therefore, the effects of different NR treatments should be explored.

IR‐induced HSC senescence is related to a remarkable increase in ROS level, and HSCs display ROS‐dependent long‐term hematopoietic defects after exposure to modest doses of IR, which are partly regulated by multiple oxidative stress pathways (Henry et al., 2020). However, our previous study showed that the inhibition of p38 MAPK insufficiently attenuated the senescence of HSCs triggered by TBI in mice (Lu et al., 2016). In addition to intracellular amino acid deficiency, GCN2/eIF2α/ATF4 signal pathway is also activated by oxidative stress. The GCN2/eIF2α/ATF4 pathway can be activated by mitochondria‐derived ROS in gastric cancer cells to increase GSH production (Wang et al., 2018). In addition, ROS‐mediated GCN2/eIF2α activation partially contributes to the microcystin‐leucine‐arginine‐mediated downregulation of steroidogenic proteins and synthases (Gao et al., 2021). Our results showed that IR increased intracellular ROS levels and stimulated the GCN2/eIF2α/ATF4 axis in LSKs, whereas NR treatment restrained oxidative stress and inhibited activation of the GCN2/eIF2α/ATF4 signaling pathway in LSKs of IR‐induced premature aging mice. The combination of natural polyphenols with NR and a TLR2/6 ligand, lipopeptide, protected mice against lethal γ radiation (Obrador et al., 2022). To improve therapeutic effects, a combination of NR with other agents might be better for mitigating IR‐induced DEARE. Loss of genetic information resulting from DNA damage, particularly double‐stranded DNA breaks, is the primary factor contributing to the aging process. A recent study posits that the degeneration of epigenetic molecules is the primary cause of aging (Yang et al., 2023). Therefore, it is imperative to investigate the impact of IR on the epigenetic state of HSCs. Although this cannot be achieved with current cohorts, it will soon be accomplished.

## 5 CONCLUSION

In conclusion, our study demonstrates that TBI accelerates the aging process in mice. Administration of NR assists senescent spleen recovery in IR‐induced premature aging mice and ameliorated serum metabolism profiles. Further results demonstrated that NR treatment alleviated LSKs senescence probably by mitigating the ROS‐activated GCN2/eIF2α/ATF4 pathway in mice with IR‐induced premature aging. Therefore, NR may be used as a DEARE therapeutic agent for patients receiving radiotherapy and radiation‐exposed populations.

## AUTHOR CONTRIBUTIONS

Wenxuan Li: performed, analyzed the experiments and wrote the manuscript; Xinyue Wang, Yinping Dong, Qidong Huo, Xin Wu, and Yinping Dong: performed and analyzed experiments; Lu Lu, Junling Zhang, and Yu Zhao: visualization and writing—review and editing the manuscript; Hui Dong and Deguan Li: conceptualization, funding acquisition, resources, supervision and writing—review and editing.

## CONFLICT OF INTEREST STATEMENT

The authors have declared that no competing interest exists.

## PERMISSION STATEMENT

The study has been approved by the Ethics Committee of the IRM‐CAMS (No. IRM‐DWLL‐2021024).

## Supporting information


Data S1

## Data Availability

All data in the study are available.
