# Supplementary material for: Nicotinamide riboside intervention alleviates hematopoietic system injury of ionizing radiation‐induced premature aging mice
Source: Aging Cell. 2023 Aug 31;22(11):e13976. doi: 10.1111/acel.13976 (PMC10652312; doi:10.1111/acel.13976)
Supplement: Supplementary file 1 — Data S1 [file ACEL-22-e13976-s001.docx]

**Supplementary materials**

**Table S1. Antibodies used in this study**

| Antibodies | CAT | Source |
| --- | --- | --- |
| Anti-mouse Ly-6G Brilliant Violet 785 | 127645 | BioLegend |
| Anti-mouse Ly-6C Alexa Fluor 488 | 128022 | BioLegend |
| Anti-mouse CD170 eFluor 660 | **50-1702-82** | eBioscience |
| Anti-mouse CD115 Super Bright 600 | 63-1152-12 | eBiosciece |
| Anti-mouse CD11b Super Bright 645 | 64-0112-82 | eBiosciece |
| Anti-mouse CD172a PerCP-eFlour 710 | 46-1721-82 | eBiosciece |
| Anti-mouse CD45 APC-eFluor 780 | 47-0451-82 | eBiosciece |
| Anti-mouse CD3 APC | 17-0032-82 | eBiosciece |
| Anti-mouse CD45R/B220 PerCP/Cyanine5.5 | 103236 | BioLegend |
| Anti-mouse CD11b eFluor 450 | 48-0112-82 | eBiosciece |
| Anti-mouse Ly-6G/Ly-6C (Gr-1) eFluor 450 | 48-5931-82 | eBiosciece |
| Anti-mouse Lineage Panel Biotin | 133307 | BioLegend |
| Streptavidin Brilliant Violet 650 | 405232 | BioLegend |
| Anti-mouse CD117 (c-kit) APC | 135108 | BioLegend |
| Anti-mouse Ly-6A/E (Sca-1) APC-Cy7 | 108126 | BioLegend |
| Anti-mouse CD34 PE/Dazzle 594 | 128616 | BioLegend |
| Anti-mouse CD16/32 BV421 | 562896 | BD Bioscience |
| Anti-mouse CD127 PerCP/Cyanine5.5 | 135022 | BioLegend |
| Anti mouse CD135 APC | 560718 | BD Bioscience |
| Streptavidin APC-Cy7 | 554063 | BD Bioscience |
| Anti-mouse CD117 (c-kit) PE-Cy5 | 15-1171-83 | eBiosciece |
| Anti-mouse Ly-6A/E (Sca-1) PE-Cy7 | 25-5981-82 | eBiosciece |
| Anti-mouse CD45.1 FITC | 110706 | BioLegend |
| Anti-mouse CD45.2 PE | 109808 | BioLegend |
| Anti-mouse phospho-GCN2 (Thr898) | BS3155R | Bioss |
| Anti-mouse p-EIF2S1(Ser51) CoraLite Plus 488 | CL488-68023 | Proteintech |
| Anti-mouse ATF4 | 10835-1-AP | Proteintech |
| Anti-mouse GAPDH | 60004-1-Ig | Proteintech |
| Anti-mouse p38 MAKP | 14064-1-AP | Proteintech |
| Anti-ACTIVE p38 antibody | # V1211 | Promega |
| Anti-mouse p53 | 60283-2-Ig | Proteintech |
| Anti-mouse SIRT1 | 8469S | Cell Signaling Technology |
| Anti-mouse p16 ^INK4A^ | 29271S | Cell Signaling Technology |
| Anti-mouse p21 ^CIP1^ | sc-6246 | Santa Cruz Biotechnology |
| Anti-rabbit IgG H&L Alexa Fluor 555 | ab150078 | Abcam |
| Anti-rabbit IgG H&L Alexa Fluor 488 | ab150077 | Abcam |
| Anti mouse-IgG HRP | SA00001-1 | Proteintech |
| Anti rabbit-IgG HRP | SA00001-2 | Proteintech |

**Table S2. Primers used for quantitative PCR in this study.**

| Gene | Forward | Reverse |
| --- | --- | --- |
| p38 MAPK | GCCACCGATGGACCTCAAG | GTCCTCCAAAAGTGTGGGAGA |
| p16 ^INK4a^ | CGCAGGTTCTTGGTCACTGT | TGTTCACGAAAGCCAGAGCG |
| SIRT1 | GCTGACGACTTCGACGACG | TCGGTCAACAGGAGGTTGTCT |
| p53 | TGGAAGGAAATTTGTATCCCGA | GTGGATGGTGGTATACTCAGAG |
| p21 ^CIP1^ | CCTGGTGATGTCCGACCTG | CCATGAGCGCATCGCAATC |
| GAPDH | ATGACATCAAGAAGGTGGTG | CATACCAGGAATGAGCTTG |


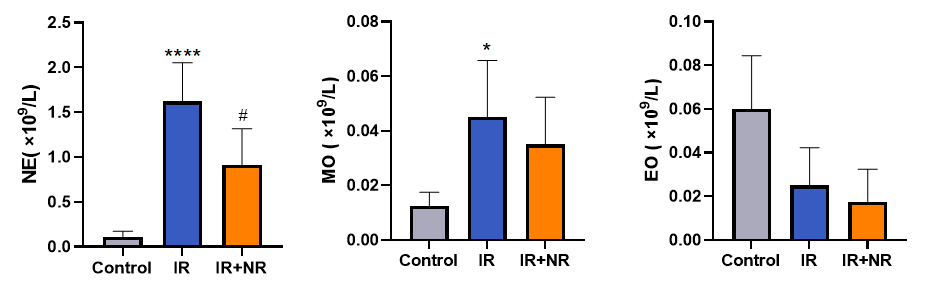
**Figure S1**

Figure S1. The numbers of neutrophils, monocytes and eosinophils in peripheral blood (n=4).
